# A personalized mHealth intervention for the universal prevention of perinatal mental disorders in routine maternal care (e-Perinatal): A protocol study for a hybrid feasibility pilot trial

**DOI:** 10.12688/openreseurope.20386.1

**Published:** 2025-06-27

**Authors:** Francisco J. Nieto-Casado, Irene Gómez-Gómez, Ignacio Aznar-Lou, Carlos Barquero-Jiménez, Isabel Cáceres, Stephanie Carretero, Rosalba Company-Córdoba, Paula De-Juan-Iglesias, Sara Domínguez-Salas, José J. Gil-Cosano, Lennert Goossens, Carmen Rodríguez-Domínguez, Emma Motrico

**Affiliations:** 1 Departamento de Psicología Evolutiva y de la Educación, Universidad de Sevilla, Seville, 41018, Spain; 2 Instituto de Biomedicina de Sevilla, IBiS/Hospital Universitario Virgen del Rocío/CSIC/Universidad de Sevilla, Seville, 41013, Spain; 3 Departamento de Psicología, Universidad Loyola Andalucía, Seville, 41704, Spain; 4 Health Technology Assessment in Primary Care and Mental Health, Institut de Recerca Sant Joan de Déu, Barcelona, 08950, Spain; 5 Centro de Investigación Biomédica en Red de Epidemiología y Salud Pública, Madrid, 28029, Spain; 6 Departamento de Psicología Experimental, Universidad de Sevilla, Seville, 41018, Spain; 7 Department of Health and Biomedical Sciences, Universidad Loyola Andalucía, Seville, 41704, Spain; 8 Departamento de Psicología Social, Universidad de Sevilla, Seville, 41018, Spain

**Keywords:** Perinatal mental disorders, prevention, mHealth, randomised controlled trial, feasibility, acceptability, preliminary effectiveness

## Abstract

**Introduction:**

The perinatal period is associated with an increased risk of new-onset depression and anxiety in both mothers and their partners. Despite the significant impact of perinatal mental disorders (PMD) on families and healthcare systems, access to mental health services remains limited due to structural barriers. Mobile health (mHealth) interventions offer a scalable and accessible strategy for universal prevention, but few have been integrated into routine maternal care and evaluated in real-world settings.

**Protocol:**

This study presents the protocol for a two-arm, cluster-randomised, hybrid type 1 pilot trial designed to evaluate the feasibility, acceptability, and preliminary effectiveness of the e-Perinatal app, a personalised mHealth innovation for universal PMD prevention. Eight Primary Healthcare Centres in Andalusia, Spain, will be randomised in a 1:1 ratio to the intervention or control arm. The intervention group will receive access to the e-Perinatal app along with specialised training in perinatal mental health for healthcare professionals involved in routine maternal care. The control group will receive routine maternal care along with monthly psychoeducational emails. A total of 96 pregnant or postpartum women (up to five months postpartum) and their partners will be recruited. Primary outcomes are feasibility and acceptability of the e-Perinatal app as a universal preventive intervention integrated within routine maternal care. Secondary outcomes include implementation measures (i.e., adoption, fidelity, and appropriateness) and preliminary effectiveness indicators (i.e., cumulative incidence of perinatal depression and anxiety, changes in symptoms severity, postnatal post-traumatic stress disorder, and subjective well-being). Additional outcomes include other implementation measures (e.g., participants experiences and reasons to dropout), family dynamics, infant development, app usability, and healthcare utilisation. Data will be analysed using mixed methods.

**Discussion:**

This pilot trial will provide key information on the feasibility of integrating a personalised mHealth intervention into routine maternal care, thereby informing the design of a subsequent large-scale trial.

## Introduction

The perinatal period, spanning from pregnancy to the first year postpartum, is a time of profound neuropsychological, physiological, and social change ( Hoekzema *et al*., 2020). While this life stage brings opportunities for growth and adjustment, it also increases vulnerability to mental health disorders ( WHO, 2015). Among these, perinatal depression and anxiety are particularly prevalent, with meta-analyses estimating that approximately 15% to 20% of women experience clinically significant symptoms during this period ( Dennis *et al*., 2017; Hahn-Holbrook *et al*., 2018). In fathers, prevalence rates are slightly lower (around 10%) but still represent a relevant public health concern ( Leach *et al*., 2016; Rao *et al*., 2020).

The impact of perinatal mental disorders (PMD) extends beyond individual distress and daily functioning, adversely affecting family dynamics, as well as child development and well-being ( Gentile & Fusco, 2017; Philpott *et al*., 2020; Stein *et al*., 2014). Longitudinal studies and meta-analyses have shown that children exposed to parental PMD are at increased risk for emotional dysregulation, insecure attachment, and developmental difficulties that may persist into adolescence ( Rogers *et al*., 2020; Slomian *et al*., 2019; Sweeney & MacBeth, 2016; Wu *et al*., 2024).

In addition to these psychosocial effects, PMD place a considerable economic burden on healthcare systems and society. Emerging evidence indicates that untreated perinatal depression and anxiety are associated with reduced work productivity, income loss, increased utilisation of healthcare services, and long-term costs related to child health and developmental issues ( Bauer *et al*., 2016; Platt *et al*., 2024).

Despite growing recognition of their prevalence and consequences, parental PMD remain underdiagnosed and undertreated in many health systems around the world ( Fonseca *et al*., 2020; O’Connor *et al*., 2019). Structural barriers, such as limited availability of mental health professionals in primary care, along with persistent stigma surrounding perinatal mental health, continue to restrict access to appropriate services ( Bina *et al*., 2024; Daehn *et al*., 2022; Marshman *et al*., 2023). Although pharmacological and psychotherapeutic treatments have been shown to be effective in reducing existing PMD ( Cuijpers *et al*., 2008; Molenaar *et al*., 2018), these interventions often fail to address the high incidence of new-onset depressive and anxiety disorders that frequently occur throughout the perinatal period ( Muñoz, 2019).

As a result, increasing attention is being directed towards the development and implementation of universal preventive approaches within perinatal care settings ( O’Connor *et al*., 2019; WHO, 2022). Universal preventive interventions aim to reduce the incidence of depressive and anxiety disorders in all pregnant and postpartum women and their partners, regardless of their individual risk of developing such conditions ( Arango *et al*., 2018; WHO, 2022). By targeting individuals without a clinical diagnosis, these interventions seek to promote protective factors and reduce risk factors before significant clinical symptomatology emerges ( Cuijpers, 2023).

Advantages of universal interventions include reduced stigma, since all individuals receive the same preventive measures, and improved feasibility for large-scale implementation, as providers are not required to conduct exhaustive screening to identify specific at-risk groups ( Cuijpers, 2023; Yasuma *et al*., 2020). Nevertheless, despite these potential benefits, the effectiveness of universal interventions remains largely theoretical and has not been consistently supported by empirical evidence. Indeed, previous randomised controlled trials (RCT) exploring universal prevention for PMD have yielded mixed or modest results, often hindered by high dropout rates and limited integration into routine clinical practice (e.g., Motrico *et al*., 2023).

In recent years, digitally delivered interventions have emerged as a promising solution to many of the barriers associated with traditional face-to-face preventive interventions. With the widespread availability of smartphones, even among individuals in low-income and rural areas ( International Telecommunication Union, 2010), mobile health (mHealth) interventions offer a scalable method to support mental health. These interventions require minimal synchronous professional presence while remaining professionally guided, thereby alleviating resource constraints that often limit access to perinatal mental health services ( Deady *et al*., 2017; Muñoz *et al*., 2018; Rigabert *et al*., 2020).

Despite these advantages, the implementation of mHealth interventions into perinatal routine care remains limited due to concerns about privacy and data security, as well as structural barriers such as time constraints and insufficient training among healthcare professionals ( Borghouts *et al*., 2021; Giebel *et al*., 2023; Webb *et al*., 2021; Webb *et al.,* 2024). In addition, most existing mHealth interventions primarily focus on maternal mental health and rarely involve partners ( de-Juan-Iglesias *et al*., 2024; Shorey *et al*., 2019). Furthermore, many commercially available applications lack empirical validation, making their effectiveness in improving perinatal mental health outcomes uncertain ( Zeng *et al*., 2023; Zhou *et al*., 2022). These limitations underscore the urgent need for rigorously developed and systematically evaluated mHealth interventions that are tailored to the needs of both mothers and partners, while ensuring feasibility and implementation into routine maternal care.

To bridge this gap, it is essential to adopt conceptual and theoretical approaches that guide both the design of effective preventive interventions and their sustainable implementation in clinical practice. The development of mHealth interventions requires a systematic methodology, such as that outlined by the Medical Research Council framework for complex interventions ( Skivington *et al*., 2021). This framework provides guidance on how complex interventions should be designed, piloted, and evaluated to ensure their effectiveness and scalability in real-world settings.

Additionally, the Normalization Process Theory (NPT) offers a robust theoretical lens for understanding how complex interventions can be integrated into routine care practices ( May, 2006; May *et al*., 2009). The NPT posits that for an innovation to become “normalised”, stakeholders must understand the intervention (i.e., coherence component), commit to it (i.e., cognitive participation component), incorporate it into their work (i.e., collective action component), and reflect on its impact (i.e., reflexive monitoring component). In the context of perinatal mental health, applying this theory helps to identify potential barriers against and facilitators for intervention adoption. It also supports the development of digital interventions that are grounded in clinical realities and responsive to the needs of both healthcare professionals and service users ( May *et al*., 2018).

In response to the need for scalable and evidence-based digital preventive interventions, the present study aims to evaluate the feasibility and acceptability of the e-Perinatal app, an mHealth intervention designed for the universal prevention of PMD within routine maternal care in the Andalusian regional public healthcare system in Spain. The primary objective of this pilot is to examine feasibility outcomes, including recruitment, retention, and adherence patterns among pregnant and postpartum women and their partners. These findings will inform the design of a subsequent large-scale RCT to determine the effectiveness, cost-effectiveness, and implementation of the intervention. As secondary objectives, the study also seeks to assess the acceptability of the e-Perinatal app among pregnant and postpartum women, their partners (i.e., fathers and non-birthing parents), and healthcare professionals involved in routine maternal care. Furthermore, it aims to evaluate the preliminary effectiveness of the intervention in reducing the incidence of new-onset perinatal depressive and anxiety disorders among women, while also examining its potential to alleviate symptom severity in partners. Potential benefits for infant developmental outcomes will also be explored.

## Protocol

### Design

This study is a pilot, two-arm, cluster-RCT with a hybrid type 1 design. Clusters will be represented by primary healthcare centres (PHC), which will be pre-randomised to either the intervention or control arm in a 1:1 allocation ratio. The study protocol adheres to the Standard Protocol Items: Recommendations for Interventional Trials (SPIRIT) guidelines ( Butcher *et al*., 2022; Chan *et al*., 2013).

The trial was registered on ClinicalTrials.gov (NCT06640907) on October 11, 2024, prior to the start of participant recruitment. The complete trial record is available at https://clinicaltrials.gov/study/NCT06640907.

### Study setting

This trial will be conducted within the routine maternal care services of the Andalusian Health Service (Servicio Andaluz de Salud, SAS) in Spain. The regional healthcare system in Andalusia is publicly funded, ensuring universal access to perinatal care through the primary care network, where PHC serve as the first point of contact for pregnant and postpartum women and their partners.

Routine maternal care in this setting follows the standard practices of the Spanish National Health System, adapted regionally ( Consejería de Igualdad, Salud y Políticas Sociales, 2014). Pregnant women are offered ten scheduled midwife visits at different gestational weeks, complemented by one postnatal midwife visit approximately two weeks after childbirth. Infants receive six routine check-ups with nurses and paediatricians during their first year of life. In addition, PHC provide access to childbirth education programmes free of charge, typically delivered as group sessions covering pregnancy, childbirth, postpartum recovery, and newborn care.

Healthcare is delivered by a multidisciplinary team comprised of midwifes, nurses, paediatricians, and other professionals responsible for maternal and infant health monitoring, health education, and the implementation of preventive interventions. The integration of the e-Perinatal intervention into this network aims to enhance existing care pathways through digital and evidence-based support, while also reinforcing healthcare professionals’ competences in perinatal mental health.

### PHC and participants

This study focuses on women and their partners during the perinatal period. Participants will be recruited from eight PHC in the province of Seville, Spain, intentionally selected to ensure geographic and demographic diversity, including both urban and non-urban areas with varying socioeconomic and cultural backgrounds.

At the PHC level, eligibility criteria include (a) ensuring the PHC manager signs the collaboration agreement and adheres to ethical standards and (b) securing the agreement and informed consent of at least one midwife and/or paediatrician to participate in the study. There are no exclusion criteria for PHC.

At the participant level, women will be eligible to participate if they (a) receive an invitation from a healthcare professional at one of the participating PHC, (b) are at least 16 weeks pregnant or within five months postpartum at the time of enrolment, (c) are at least 18 years old, (d) have access to a mobile phone and to internet, (e) are able to read, write, and understand Spanish, and (f) have a personal email account. Women will be excluded if they (a) meet diagnostic criteria for anxiety or depression based on the Mini-International Neuropsychiatric Interview (MINI, Sheehan *et al*., 1998) or (b) are on a waiting list or currently receiving psychological or pharmacological treatment for any mental health or substance use condition.

Partners are defined as the individual identified by the participant woman as her main support during the perinatal period. This may include a spouse—regardless of gender—or another significant family member. Partners will be eligible to participate if they (a) receive an invitation from a woman already enrolled in the study, (b) are at least 18 years old, (c) have access to a mobile phone and to internet, (d) are able to read, write, and understand Spanish, and (e) have a personal email account. There are no exclusion criteria for partners. Partners may withdraw from the study at any time without affecting the woman’s participation or the routine maternal care she receives at the PHC. Conversely, partners may continue in the study even if the woman withdraws.

### Intervention

***Intervention arm.*** The intervention arm of this study involves a comprehensive approach that combines the e-Perinatal app with targeted training for healthcare professionals to support the prevention of PMD within routine maternal care settings. Participants allocated to this arm will use the e-Perinatal app for a period of two months.

The e-Perinatal app is an mHealth intervention grounded in evidence-based efforts for the prevention of perinatal depression and anxiety. Its base content includes psychoeducational materials, psychological strategies, mindfulness practices, and physical activity resources, all tailored to the needs of pregnant and postpartum women and their partners. This content is organised into thematic blocks covering key areas such as mental health, physical activity, sleep and diet, parent-infant bonding, breastfeeding, co-parenting, and partner support.

Within each thematic block, content is delivered through digital micro-interventions (DM), defined as brief and targeted components designed to be self-directed and easily integrated into daily life ( Baumel *et al*., 2020). These DM are available in text, podcast, and video formats. Text-based content can be read in under three minutes and includes a summary of key points. Podcast and video content, focusing respectively on mindfulness and physical activity, ranges from 3 to 30 minutes in duration.

A clinical rule-based filtering algorithm tailors DM recommendations based on user-provided data, including pregnancy or postpartum stage, participant role (i.e., mother or partner), individual risk factors for PMD (e.g., medical contraindications, twin pregnancy, or previous children), and content preferences. Based on these inputs, the app will display personalised content and send weekly notifications with three recommended DM. Nonetheless, users will be able to adjust the frequency of notifications and will have full access to the entire library of DM at any time.

In addition to personalised content, the app offers several features to enhance user support. This includes a resource repository linking to relevant local and online services, a discussion forum where participants can share experiences and ask questions, and a chat function staffed by psychologists. Through the chat, users can submit questions and receive responses within 48 hours. Although the app is not intended for crisis intervention, it provides clear guidance for participants experiencing crises, encouraging them to seek specialised support and offering contact information for local emergency and mental health hotline numbers. A detailed description of the development and structure of the e-Perinatal app is available in Company-Córdoba *et al*. (2025).

In parallel with the use of the app, this arm includes a structured training programme for healthcare professionals working in the participating PHC. Prior to the start of the trial, informational meetings will be held with midwives, nurses, and other healthcare professionals from both the intervention and control PHC to introduce the study objectives, procedures, and recruitment method. All participating professionals will sign a formal collaboration agreement, confirming their commitment to the study.

Professionals in the intervention arm will receive specialised training aimed at enhancing their knowledge and skills in perinatal mental health, digital preventive interventions, and the integration of the e-Perinatal app into routine maternal care. The training will cover the importance of PMD prevention, assessment tools for identifying risk factors, evidence supporting the use of digital tools for mental health promotion, and a detailed overview of the components and features of the e-Perinatal app.

This training will be delivered through a mixed format that includes one in-person session and seven online sessions (20 hours in total). The programme will be officially accredited by the Andalusian Health Service, with professionals receiving recognised certification upon completion. Healthcare professionals will also be provided with a training manual and a digital recruitment logbook to facilitate participant enrolment and monitoring. During the implementation phase, a dedicated recruitment coordinator will maintain regular contact with healthcare professionals via telephone and email to support the integration of the app into routine maternal care and to assist in resolving implementation challenges.

***Control arm.*** Participants in the control group will receive standard maternal care as routinely provided by the Andalusian Health Service. Unlike those in the intervention group, they will not have access to the e-Perinatal app or any of its intervention components. Instead, and to maintain engagement throughout the study period (two months), participants in the control arm will receive monthly emails containing psychoeducational resources on perinatal mental health, pregnancy, and infant development. These materials, developed and publicly distributed by the Andalusian Health Service, provide general information without personalised content.

Healthcare professionals in PHC assigned to the control arm will be informed about the aims, procedures, and recruitment strategies of the study. They will attend the initial meetings and more general training sessions but will not receive specific training in perinatal mental health or in the use of digital tools. This approach ensures that routine maternal care provided in these PHC remains unchanged, allowing for a clear comparison between standard routine care and the intervention.

As in the intervention arm, professionals in the control arm will receive the training manual and the digital recruitment logbook. They will also have access to the recruitment coordinator, ensuring consistent support and communication throughout the implementation phase.

### Outcomes

Participants in both the intervention and the control arms will be assessed at four key timepoints: enrolment (t _-1_), baseline (t _0_; one day after enrolment), postintervention (t _1_; two months after baseline), and follow-up (t _2_; one month after postintervention). Outcome measures include primary, secondary, and additionally exploratory outcomes. A summary of all study instruments, including the respondent type (i.e., healthcare professionals, pregnant and postpartum women, or partners) and their corresponding timepoints, is provided in Table 1.

**Table 1.** Overview of study instruments and corresponding assessment timepoints.

Instruments	Measures	Before recruitment	Screening (t _-1_)	Baseline (t _0_)	Postintervention (t _1_)	Follow-up (t _2_)
Organizational Readiness Implementing Change (ORIC) _a_	Organizational readiness to implement the intervention	X				
Normalization Measure Development Questionnaire (NoMAD) _a_	Implementation process and normalization in routine perinatal care				X	
User Version of the Mobile Application Rating Scale (uMARS) _b_	App quality				X	
Mini International Neuropsychiatric Interview (MINI) _c d_	Maternal perinatal depression and maternal perinatal anxiety		X		X	
Edinburgh Postnatal Depression Scale (EPDS)	Depressive symptoms		X	X	X	X
General Anxiety Questionnaire (GAD-7)	Anxiety symptoms		X	X	X	X
City Birth Trauma Scale (City-BiTS) _c e_	Postnatal post-traumatic stress			X		
Well-Being Index (WHO-5)	Psychological well-being			X	X	X
Antenatal Risk Questionnaire (ANRQ-R)	Antenatal/postnatal risk			X		
Maternal Antenatal Attachment Scale (MAAS) _d_	Maternal antenatal attachment			X		
Paternal Antenatal Attachment Scale (PAAS) _f_	Paternal antenatal attachment			X		
Maternal Postnatal Attachment Scale (MPAS) _c_	Maternal postnatal attachment			X		
Paternal Postnatal Attachment Scale (PPAS) _e_	Paternal postnatal attachment			X		
Ages & Stages Questionnaires (ASQ-3) _c_	Infant development			X		
Infant Behavior Questionnaire-Revised Short Form (IBQ-R SF) _c_	Infant temperament			X		
Breastfeeding ( *ad hoc*) _c_	Infant feeding practices and satisfaction with breastfeeding			X		
Coparenting Relationship Scale (CRS) _c e_	Coparenting dynamics			X		
Parental Sense of Competence scale (PSOC) _c e_	Parental role perception			X		
Quality of Marriage Index (QMI)	Partnership quality			X		
Pittsburgh Sleep Quality Index (PSQI)	Sleep quality			X		
European Quality of Life 5-Dimensions (EuroQol-5D-3L)	Quality of life			X		
Pregnancy Physical Activity Questionnaire (PPAQ) _c d_	Physical activity during pregnancy and postpartum			X		
International Physical Activity Questionnaire (IPAQ) _e f_	Physical activity in partners			X		
International Fitness Scale (IFIS)	Physical fitness			X		
Positive and Negative Affect Schedule (PANAS)	Mood state			X		
Basic Psychological Need Satisfaction and Frustration Scale – Short Form (BPNSFS)	Satisfaction and frustration of basic psychological needs			X		

*Note*. _a_ = Instrument completed by healthcare professionals; _b_ = Instrument completed only by participants of the intervention arm; _c_ = Instrument completed only by postpartum women; _d_ = Instrument completed only by pregnant women; _e_ = Instrument completed only by partners of postpartum women; _f_ = Instrument completed only by partners of pregnant women.

***Sociodemographic.*** A structured questionnaire will be used to gather sociodemographic information, including gender, age, educational level, socioeconomic status, working status, marital status, and number of previous children. For pregnant and postpartum women, additional items will assess relevant obstetric factors, such as complications during pregnancy and medical contraindications during the perinatal period. Information related to the child (e.g., sex, expected due date or actual date of birth, and gestational week at birth) will also be collected.

***Primary outcomes.*** The primary outcomes of this study are the feasibility and acceptability of the e-Perinatal app as a universal preventive intervention for perinatal depression and anxiety, integrated in routine maternal care. Assessing these outcomes is critical to determine the viability of implementing the intervention in real-world healthcare settings and to guide the design of a subsequent large-scale trial.

Feasibility will be assessed through recruitment, participation, and retention rates ( Pearson *et al*., 2020). Recruitment rate will be determined by the proportion of eligible women and partners who provide consent to enrol in the study, while participation rate will be calculated based on the number of participants successfully included in the pilot. Retention will be measured by the proportion of participants who complete the follow-up assessment. These indicators will be extracted from enrolment and participant records and assessed at t _0_ and t _1_.

Acceptability will be assessed in two stages, early acceptability and final acceptability ( Eldridge *et al*., 2016b). Early acceptability will be determined by the number of eligible women and partners who consent to participate, reflecting initial willingness to engage with the intervention. Final acceptability will be assessed after participants have used the e-Perinatal app, evaluating their perspectives on the appropriateness, satisfaction, and feasibility of the intervention. This will be measured using a combination of structured surveys and semi-structured interviews based on the NPT ( May, 2006; May *et al*., 2009). The surveys will assess user satisfaction and perceived usability of the app, while the qualitative interviews will provide in-depth understanding of barriers, facilitators, and overall experience of participants. Final acceptability will be assessed at t _1_.

***Secondary outcomes.*** The secondary outcomes of this study address both key implementation and effectiveness indicators of the e-Perinatal app.

Implementation outcomes include adoption, fidelity ( Moore *et al*., 2015), and appropriateness ( Weiner *et al*., 2017). Adoption will be evaluated by determining the proportion of healthcare professionals who express willingness to participate in the study prior to the start of the intervention ( Glasgow *et al*., 1999; Glasgow *et al*., 2001). This measure collects the initial level of professional engagement and interest in integrating the e-Perinatal app into the usual maternal care practices. Adoption rates will be extracted from registration records before participant recruitment begins.

Fidelity refers to the degree to which healthcare professionals adhere to the study protocol and recommended implementation procedures ( Bellg *et al*., 2004). This will be measured using ad hoc structured surveys completed by these professionals at t _1_.

Appropriateness will also be examined in two phases, early appropriateness and final appropriateness. Early appropriateness will be measured by the number of eligible women invited to participate and the number of those who extend invitations to their partners. This will be assessed using recruitment records and consent forms at t _-1_. Final appropriateness will focus on evaluating the suitability and relevance of the intervention for the target population and healthcare setting. This will be assessed through semi-structured interviews based on the NPT ( May, 2006; May *et al*., 2009), measuring the perspectives of both participants and healthcare professionals at t _1_.

Effectiveness outcomes include the cumulative incidence of perinatal depression and anxiety in participant women, changes in depressive and anxiety symptoms during the intervention, postnatal post-traumatic stress, and subjective well-being.

The cumulative incidence of perinatal depression and anxiety among pregnant and postpartum women will be measured using the Mini International Neuropsychiatric Interview (MINI; Sheehan *et al*., 1998), a structured diagnostic interview comprised of 14 modules. In this study, only six modules will be administered via telephone: (1) major depressive episode, (2) panic disorder, (3) agoraphobia, (4) social anxiety disorder, (5) post-traumatic stress disorder, and (6) generalised anxiety disorder modules.

Changes in depressive symptoms in all participants will be measured using the Edinburgh Postnatal Depression Scale (EPDS; Cox *et al*., 1987; Garcia-Esteve *et al*., 2003), a 10-item self-report instrument specifically designed to screen for perinatal depression. Participants rate each item on a 4-point Likert scale (0–3), with higher scores indicating greater depressive symptom severity.

Changes in anxiety symptoms in all participants will be evaluated using the Generalized Anxiety Disorder Scale (GAD-7; García-Campayo *et al*., 2010; Spitzer *et al*., 2006), a 7-item self-report measure assessing the severity of anxiety symptoms. Each item is scored on a 4-point Likert scale (0–3), with higher scores indicating more pronounced anxiety symptoms.

Postnatal post-traumatic stress will be assessed through the City Birth Trauma Scale (City-BiTS; Ayers *et al*., 2018; Caparros-Gonzalez *et al*., 2021), a 29-item self-report measure developed to identify postnatal post-traumatic stress disorder (PTSD) symptoms in both postpartum mothers and their partners. Responses are scored on varying Likert scales (0–3 or 0–2), with higher scores indicating greater PTSD symptom severity.

Finally, subjective well-being in all participants will be evaluated using the World Health Organization Well-Being Index (WHO-5; WHO, 1998), a 5-item self-report scale. Each item is rated on a 6-point Likert scale (0–5), with higher scores reflecting better overall well-being.

***Other outcomes.*** This pilot study includes additional implementation and effectiveness outcomes to provide a more comprehensive evaluation of the e-Perinatal app within routine maternal care settings.

Implementation outcomes include organizational readiness for implementing change, the implementation process itself, app quality and user experience, as well as perceived barriers, facilitators, and reasons for dropout. Organizational readiness for implementing change will be assessed using the Organizational Readiness for Implementing Change scale (ORIC; Shea *et al*., 2014), a 12-item self-report measure where higher scores indicate greater readiness to adopt new interventions in clinical practices.

The implementation process of the intervention will be evaluated through the Normalization Measure Development Questionnaire (NoMAD; Finch *et al*., 2018), a 23-item self-reported instrument.

App quality and user experience will be measured using the User Version of the Mobile Application Rating Scale (u-MARS; Martin-Payo *et al*., 2021; Stoyanov *et al*., 2016), a 26-item self-reported questionnaire assessing engagement, functionality, aesthetics, information quality, and perceived impact.

Participant experiences, barriers, and facilitators influencing engagement with the intervention will be explored through semi-structured interviews based on the NPT. Reasons for dropout (e.g., loss of engagement or personal circumstances) will be collected through study records and qualitative interviews with participants who end their participation.

Effectiveness outcomes focus on evaluating the broader impact of the intervention on parental, infant, and family factors. Parental and family dynamics will be assessed using multiple validated instruments, including the Antenatal Risk Questionnaire – Revised (ANRQ-R; Reilly *et al*., 2021) to measure perinatal risk factors in both women and their partners. Attachment to the infant during pregnancy and after childbirth will be assessed using separate instruments for mothers and their partners. Maternal antenatal attachment will be measured with the Maternal Antenatal Attachment Scale (MAAS; Condon, 1993; Navarro-Aresti *et al*., 2016), while paternal antenatal attachment will be evaluated using the Paternal Antenatal Attachment Scale (PAAS; Condon, 1993). Postnatal attachment will be assessed through the Maternal Postnatal Attachment Scale (MPAS; Condon & Corkindale, 1998; Riera-Martín *et al*., 2018) and the Paternal Postnatal Attachment Scale (PPAS; Condon *et al*., 2008; Riera-Martín *et al*., 2018).

In postpartum women and their partners, coparenting dynamics will be examined using the Coparenting Relationship Scale (CRS; Feinberg *et al*., 2012), which evaluates dimensions such as cooperation, conflict, or shared responsibility in parenting. In turn, parental role perception will be measured with the Parental Sense of Competence Scale (PSOC; Johnston & Mash, 1989; Oltra-Benavent *et al*., 2020), assessing self-efficacy and satisfaction in the caregiving role.

Infant-related outcomes will include assessments of infant temperament through the Infant Behaviour Questionnaire-Revised Short Form (IBQ-R SF; Putnam *et al*., 2014; González Salinas *et al*., 2000) and evaluations of developmental milestones using the Ages & Stages Questionnaires (ASQ-3; Squires & Bricker, 2009). In addition, breastfeeding experiences will be assessed using an ad hoc instrument specifically developed by the research team for this study. This questionnaire includes items on the type of feeding provided (i.e., exclusive breastfeeding, formula feeding, or mixed feeding) and the mother’s overall satisfaction with the breastfeeding experience. These infant-related measures will be reported exclusively by birthing mothers.

Further measures will capture broader indicators of health and well-being. Sleep quality will be evaluated using the Pittsburgh Sleep Quality Index (PSQI; Buysse *et al*., 1989; Royuela & Macías, 1997). Quality of life will be measured with the European Quality of Life-5 Dimensions-3 Levels (EuroQol-5D-3L; Badia *et al*., 1999; EuroQol Group, 1990). Physical activity levels will be assessed separately for mothers and partners, using the Pregnancy Physical Activity Questionnaire (PPAQ; Chasan-Taber *et al*., 2004; Coll-Risco *et al*., 2019) for pregnant and postpartum women and the International Physical Activity Questionnaire (IPAQ; Craig *et al*., 2003; Roman-Viñas *et al*., 2010) for their partners. Additionally, physical fitness will be evaluated using the International Fitness Scale (IFIS; Ortega *et al*., 2011).

Psychological and emotional states will be assessed using multiple validated instruments. Emotional states will be evaluated with the Positive and Negative Affect Schedule (PANAS; López-Gómez *et al*., 2015; Watson *et al*., 1988). Experiences of satisfaction and frustration of the basic psychological needs (i.e., need for autonomy, competence, and relatedness) will be captured through the Basic Psychological Need Satisfaction and Frustration Scale – Short Form (BPNSFS; Chen *et al*., 2015). Perceptions of partnership quality will be measured with the Quality of Marriage Index (QMI; Norton, 1983).

Economic data will be obtained through a telephone interview at t _1_. Participants will report their use of healthcare services and sick-leave days over the previous year. Services assessed will include emergency visits, hospital admissions (including duration of each stay), primary care visits (e.g., nurse, midwife, general practitioner, and social worker), secondary care visits, and medication use, among others. All data will be classified by type of service (i.e., public or private).

### Participant timeline

During one of the scheduled routine maternal care visits, midwives or nurses at participating PHC will approach women who may be eligible to participate in the study. Those expressing interest will receive an informational flyer with details about the study and a QR code that directs them to a secure online screening platform. This platform includes a short introductory video, a detailed participant information sheet, and an electronic written informed consent form, which must be completed before any study-related procedures take place.

After providing written consent, participants will complete an initial online questionnaire to verify eligibility (t _-1_). Eligible women will then undergo the MINI interview, carried out via telephone by a psychologist from the Andalusian Health Service. This psychologist—who also serves as the recruitment coordinator—is blinded to the participant’s allocation (i.e., intervention or control arm). The purpose of this interview is to ensure that participants do not meet diagnostic criteria for depression or anxiety, which would result in exclusion from the study.

During the enrolment process on the online screening platform, women will have the option to invite their partners (or significant family member) to participate. To proceed, women will be asked to enter their partner’s email address, which will be immediately encrypted. Once the woman is formally enrolled, the partner will receive an automated email with a link to an adapted online screening platform. This platform also includes a study information sheet, an electronic written informed consent form, and an eligibility questionnaire.

Upon confirmation of eligibility, participants assigned to the intervention arm will receive an email with a link to download the e-Perinatal app and to fill out the baseline questionnaire (t _0_). They will also receive instructions on how to register and set their personalised notification preferences. Participants in the control arm will receive a similar email confirming their participation and providing a link to complete the baseline assessment (t _0_), but without access to the app.

To ensure active participation, participants in the intervention group must access the app within 15 days of receiving the email, while control group participants must complete the baseline questionnaire within the same timeframe. Participants who do not meet these requirements will be excluded from the study.

**Figure 1.**
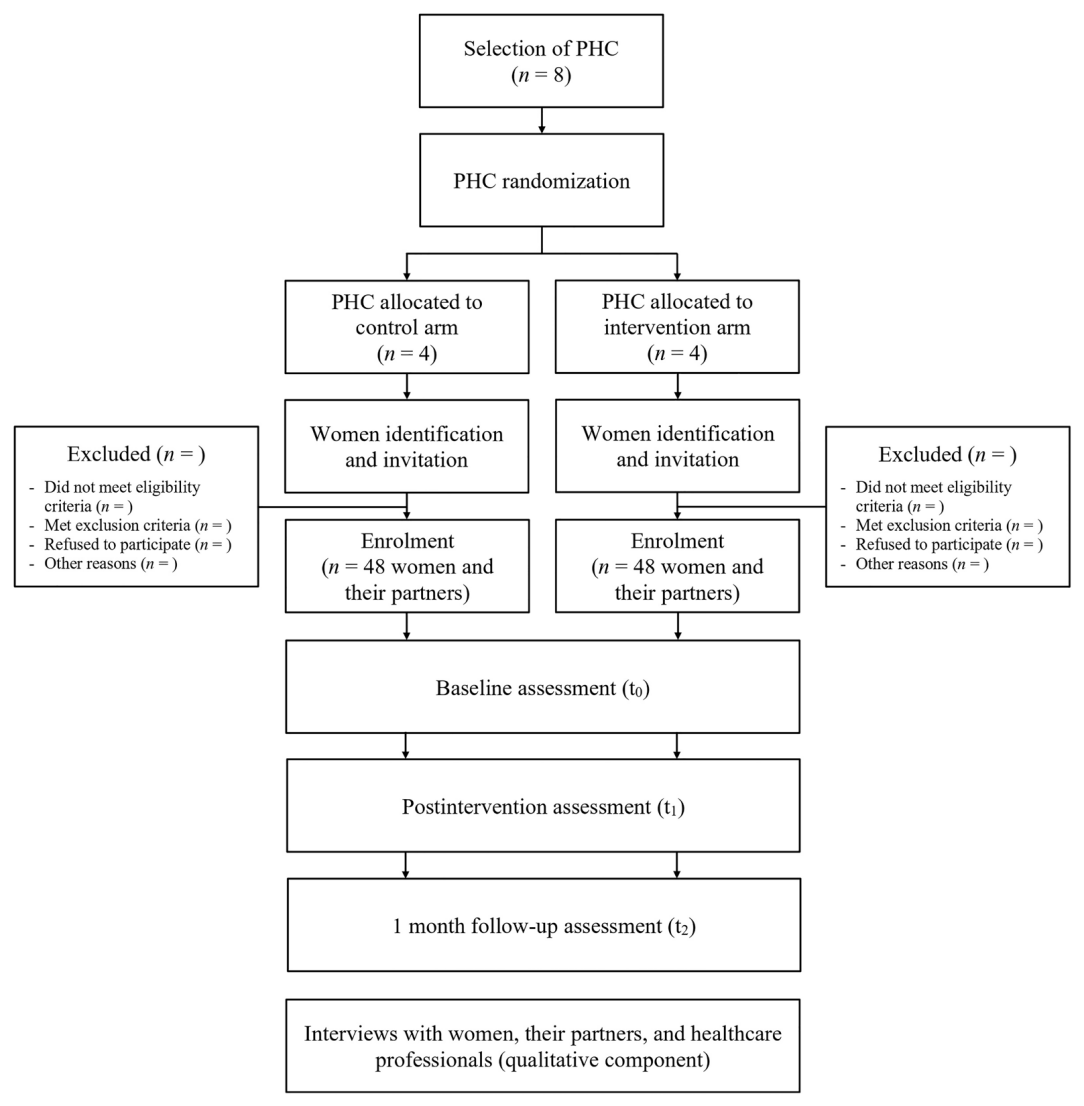
Flowchart of the study.

The overall study design and timeline are presented in Figure 1 and Table 2, respectively.

**Table 2.** Schedule of enrolment, interventions, and assessment.

	STUDY PERIOD			
	Enrolment	Intervention		Follow-up
**TIMEPOINT:**	*t _-1_*	*t _0_*	*t _1_*	*t _2_*
**ENROLMENT:**				
Eligibility screen	X			
Informed consent	X			
Allocation	X			
**INTERVENTION +** **USUAL CARE**		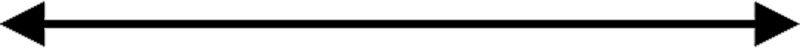		
**USUAL CARE**		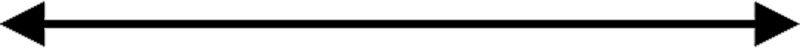		
**ASSESSMENTS:**				
Sociodemographic	X			
Maternal perinatal depression and maternal perinatal anxiety	X _a_		X _a_	
Depressive symptoms	X	X	X	X
Anxiety symptoms	X	X	X	X
Postnatal post-traumatic stress		X _c_		
Psychological well-being		X	X	X
Antenatal/postnatal risk		X		
Antenatal/postnatal attachment		X		
Partnership quality		X		
Sleep quality		X		
Quality of life		X		
Coparenting dynamics		X _c_		
Physical activity level		X		
Physical fitness		X		
Mood state		X		
Breastfeeding		X _a, c_		
Basic psychological needs		X		
Parental role perception		X _c_		
Infant development		X _a, c_		
Infant temperament		X _a, c_		
App quality			X _d_	

*Note*. _a_ = Instrument completed only by women; _b_ = Instrument completed only by pregnant women and partners; _c_ = Instrument completed only by postpartum women and partners; _d_ = Instrument completed only by the intervention group.

### Sample size

As a pilot study primarily aims to assess feasibility outcomes, no formal sample size calculation based on statistical power was conducted. Instead, the target sample size was determined pragmatically, aiming to recruit 96 pregnant and postpartum women in the second trimester of pregnancy (i.e., between gestational weeks 16 and 40) or within five months postpartum (48 per study arm). This sample size is consistent with recommendations for pilot studies ( Eldridge *et al*., 2016a; Lancaster *et al*., 2004).

The target of 96 participants was established to ensure a final sample of at least 80 women at follow-up (t _2_). This number accounts for potential dropouts and ensures sufficient data to evaluate recruitment and retention rates, adherence to the intervention, and preliminary effects of the e-Perinatal app on perinatal mental health outcomes.

As noted, women in the study will be encouraged to invite their partners to participate. Participation of partners is entirely voluntary, and up to 96 partners may join if all participating women choose to extend invitations. However, the final number of participating partners will depend on the willingness of the women to invite them.

### Recruitment

To reach the target sample size of 96 pregnant and postpartum women, contingency strategies have been established. In addition to the eight PHC initially selected to participate in the pilot, two additional PHC—one per each study arm—have been designated as reserve centres. Healthcare professionals in these PHC have completed all required training.

These reserve PHC are ready to initiate recruitment immediately if any of the initially selected centres are unable to continue or if recruitment targets are not being met (e.g., lower than expected recruitment rate). Recruitment progress will be closely monitored throughout the study, allowing the research team to activate these strategies if needed.

### Allocation

This study employs a cluster-randomised design to minimise the risk of contamination between study arms, ensuring that healthcare professionals and participants within a given PHC receive the same treatment conditions. To ensure allocation concealment, the randomisation sequence will be implemented using sealed and opaque envelopes.

Given the nature of the intervention, it is not possible to blind providers or participants to their assigned arm. Consequently, the study design will be open label with respect to participants and healthcare professionals. However, the psychologist conducting the MINI interview by telephone will be masked to the PHC allocation. This interviewer will only have access to participant ID codes and telephone numbers and will be specifically trained to avoid questions that could reveal participants’ allocation, such as inquiries about app usage or intervention-related activities. Furthermore, researchers responsible for data cleaning, preliminary checks, and statistical analyses will not have access to the randomisation codes linking PHC to their respective arms, thus minimising bias in the analysis of data.

### Data collection and management

Data will be collected at multiple levels, including PHC, healthcare professionals, and participants. All data will be stored in a secure study database, complying with data protection regulations.

At the PHC level, the research team will gather data on institutional characteristics, including the type of centre, number of healthcare professionals, and average age of service users. This information will be obtained from PHC managers using standardised electronic forms developed specifically for this study, prior to the start of participant recruitment.

At the healthcare professional level, data collection will include sociodemographic information such as gender, professional discipline, and years of experience in the PHC. Additionally, healthcare professionals will complete a recruitment logbook to track the number of women invited, the number who agreed to participate, and any relevant observation about the recruitment process. These logbooks, provided in digital format, will be collected by the research team at t _1_. Healthcare professionals will also complete questionnaires as described in the Outcomes section and will play an active role in the implementation of the intervention within routine maternal care.

At the participant level, data collection will begin with the online eligibility screening questionnaire hosted on the study’s secure screening platform. Women who meet eligibility criteria will then be scheduled for the semi-structured telephone interview using the MINI to rule out the presence of clinical depression or anxiety. Subsequent assessments (i.e., baseline, post-intervention, and follow-up questionnaires) will be completed via REDCap, a secure electronic data capture platform. Participants in the intervention arm will also complete depressive and anxiety symptom assessments directly through the e-Perinatal app, with responses securely stored in the app’s database.

In addition, a subsample of participants and healthcare professionals from the intervention arm will be invited to participate in individual semi-structured interviews at t _1_. These interviews aim to explore the implementation process at the PHC level, potential mechanisms of action, and contextual factors influencing the integration of the e-Perinatal app into routine practice. Interviews will be guided by the Normalization Process Theory (NPT; May, 2006; May *et al*., 2009) and include ad hoc questions addressing key aspects of the implementation process and barriers and facilitators to the integration of digital tools in clinical settings, considering societal, political, organisational, interpersonal, health-care professional, and individual factors ( Webb *et al*., 2021). All interviews will be audio-recorded, transcribed verbatim, and securely stored in the study database.

### Strategies for trial retention

To maximise participant retention and engagement throughout the study, a multi-faceted approach will be implemented. Participants in the intervention arm will receive a gift package containing a fit ball and resistance bands, intended to support engagement with the physical activity components of the e-Perinatal app. These items serve as both practical support for the intervention and tangible reminders of their participation in the study.

In both the intervention and control arms, participants will receive automated reminders via email to complete the online questionnaires at t _0_, t _1_, and t _2_. If an assessment is not completed within 10 days, the recruitment coordinator will contact the participant directly by telephone to provide technical support, clarify any questions about the questionnaires, and offer encouragement to continue participation.

In parallel, the recruitment coordinator will maintain regular communication with healthcare professionals at participating PHC via telephone and email. Fortnightly check-ins will provide updates on recruitment and retention rates, address any logistical challenges, and offer support to ensure participant engagement remains high.

### Potential risks for participants

As this study focuses on the prevention of perinatal depression and anxiety among low-risk or subclinical populations, the intervention is categorised as low-intensity. Low-intensity interventions are typically designed to be self-guided, non-intrusive, and supportive, requiring limited professional involvement. In this context, the e-Perinatal app primarily offers psychoeducational content aimed at promoting mental health and well-being.

The content is informational in nature and does not include prescriptive or mandatory components. Participants are free to engage with the material at their own pace and to select only those DM that are most relevant to their needs or interests. Given this flexible and user-directed design, the app poses minimal risk, even for users who interact with the content with limited interest.

Although the content is considered safe, some mental health topics or self-assessments activities may cause distress in certain participants. To mitigate this, the app includes frequent disclaimers encouraging users to seek professional help if they experience worsening symptoms or suicidal ideation. The app also offers direct access to local emergency contact information and mental health crisis hotlines.

To further protect participant safety, a structured screening procedure is conducted prior to enrolment. This includes a semi-structured diagnostic interview to ensure that only those who do not meet clinical criteria for depressive or anxiety disorders are included. If a pregnant or postpartum woman is identified as potentially meeting diagnostic criteria, she will be excluded from the study. In such cases, the interviewer will initiate a referral protocol by contacting the participant’s assigned primary care physician.

This referral protocol has been coordinated in advance with policy makers and clinical managers responsible for mental health services within the Andalusian public healthcare system. This ensures that participants requiring clinical support are directed to appropriate mental health services within the existing primary care framework.

### Data analysis

The data analysis plan for this study incorporates both quantitative and qualitative methodologies, and cross-sectional and longitudinal approaches. For baseline data, continuous variables will be summarised using means and standard deviations, while categorical variables will be reported as frequencies and percentages. Baseline differences between the intervention and control arms will be assessed using independent t-tests for continuous variables and chi-square tests for categorical variables. Given the cluster-randomised design, baseline comparability will also be evaluated at the PHC level using intraclass correlation coefficients (ICCs) to account for clustering effects. Relevant demographic variables (e.g., maternal age, educational level, marital status) and baseline measures, including depressive and anxiety symptoms (i.e., EPDS and GAD-7), will also be considered in these analyses.

For the primary outcomes, feasibility (indicated by recruitment and retention rates) and acceptability (evaluated using the u-MARS scale and qualitative interviews) will be analysed descriptively (i.e., means, standard deviations, and ranges). The transcriptions from the semi-structured interviews will be thematically analysed following the approach of Braun and Clarke (2006). In the initial coding phase, two independent researchers will read and code each transcription, identifying quotes that relate to facilitators or barriers of e-Perinatal app usage. These codes will then be organised into broad themes (e.g., “perceived ease of use” or “concerns about data privacy”). Any discrepancies between researchers will be resolved through consensus or, if needed, by the introduction of a third independent researcher.

To evaluate the incidence of maternal perinatal depression and anxiety, multilevel logistic regression models will be applied. The binary dependent variable will be the presence of new-onset depression or anxiety at postintervention (t _1_). Group allocation (i.e., intervention or control) will serve as a fixed effect, while PHC will account for random-effects parameter. The models will adjust for baseline maternal depressive and anxiety symptoms and other prognostic predictors as potential covariates. In addition, to adjust for selection bias, variables with significant baseline differences between groups will be incorporated as covariates. The results will be reported as odds ratios (ORs) with 95% confidence intervals. Additionally, the ICC will be calculated to quantify the proportion of variance attributable to clustering within PHC. To estimate absolute risk differences, the Stata “margins” command will be used, providing adjusted proportions of new depressive or anxiety episodes across the study arms.

To assess the effectiveness of the intervention in reducing depressive and anxiety symptom severity (measured by EPDS and GAD-7) among women and their partners, linear mixed-effects models with repeated measures will be employed. These models will include fixed effects for time (t _-1_, t _0_, t _1_, t _2_), group allocation, and the interaction term (time x group). Random intercepts for participants and PHC will be included to capture intra-individual and intra-cluster correlation. The models will also adjust for relevant covariates, such as maternal age, employment status, baseline symptom severity, and any unbalanced baseline variables identified during preliminary analyses.

For other continuous outcomes, such as well-being (WHO-5), mood (PANAS), and sleep quality (PSQI), the same modelling approach will be employed. For outcomes related to the child (i.e., ASQ-3 and IBQ-R SF) and for implementation measures (i.e., ORIC and NoMAD), descriptive analyses and simpler models (e.g., repeated-measures ANOVA) will be performed given the exploratory nature of the pilot.

For economic variables, mean, median interquartile range and proportion of participants with at least one use of each healthcare services will be estimated.

Finally, app engagement metrics, including the number of micro-interventions completed, total time spent, and subjective ratings of the micro-interventions’ usefulness, will be analysed descriptively.

### Dissemination

The findings from this pilot study will be disseminated through publications in peer-reviewed, high-impact scientific journals and presented at regional, national, and international conferences. All datasets and documentation resulting from this study will be made openly available via the Zenodo repository.

### Study status

At the time of first submission of this protocol, all selected PHC had been recruited, and healthcare professionals from both intervention and control arms had completed the corresponding specialised training sessions. In addition, participant recruitment and enrolment had been initiated.

## Discussion

This study presents the protocol for a pilot, two-arm, cluster-RCT with a hybrid type 1 design, aimed at evaluating the feasibility, acceptability, and preliminary implementation and effectiveness of the e-Perinatal intervention as a universal preventive solution for perinatal depression and anxiety. Given the significant incidence of new-onset depressive and anxiety disorders during pregnancy and the postpartum period ( Hahn-Holbrook *et al*., 2018; Muñoz, 2019; Rao *et al*., 2020), there is an urgent need for preventive strategies that are scalable, accessible, and adaptable to routine healthcare systems ( Fonseca *et al*., 2020; O’Connor *et al*., 2019). By integrating an evidence-based digital intervention into maternal care services, this trial aims to address persistent barriers to perinatal mental health care, such as limited availability of mental health professionals and stigma ( Bina *et al*., 2024; Daehn *et al*., 2022; Marshman *et al*., 2023), while taking advantage of the flexibility, personalisation, and cost-effectiveness of mobile health (mHealth) solutions.

A key strength of this study lies in its universal preventive approach. Rather than focusing only on at-risk individuals, the intervention is offered to the general population of pregnant and postpartum women. This approach may contribute to stigma reduction by normalising support for mental health care during the perinatal period and may improve accessibility by incorporating preventive measures within routine maternal care ( Marshman *et al*., 2023). Despite these potential advantages, empirical evidence supporting universal digital interventions for perinatal mental health remains limited. Consequently, the findings from this pilot will provide valuable data on their feasibility in real-world clinical settings.

Another important strength is the methodological rigour of the enrolment procedure. The use of the Mini-International Neuropsychiatric Interview (MINI; Sheehan *et al*., 1998), a gold-standard structured diagnostic interview, ensures the accurate identification and exclusion of women who meet diagnostic criteria for depression or anxiety. This guarantees that the intervention is tested in an appropriate preventive context, avoiding clinical cases and reinforcing its internal validity.

The study also adopts a hybrid type 1 design, which allows for the simultaneous evaluation of implementation and effectiveness outcomes. By assessing measures such as adoption, appropriateness, fidelity, and acceptability, this trial will provide significant information about the factors and processes that facilitate or hinder the integration of digital interventions into routine maternal care. This approach is in line with current best practices for complex intervention research ( Skivington *et al*., 2021) and may facilitate the translation of evidence into routine care practices ( May, 2006; May *et al*., 2009).

In addition, the trial is embedded into existing public maternal healthcare services in Andalusia, Spain. This real-world setting enhances the external validity of the findings and supports the long-term goal of integrating digital preventive interventions into national and international health systems. By setting the intervention in a publicly funded care system, the study incorporates routine clinical conditions and involves professionals already responsible for maternal and infant care, increasing the likelihood of sustainable implementation.

Furthermore, the study design employs a cluster-randomised approach to minimise the risk of contamination. Given that healthcare professionals play an important role in participant recruitment and intervention delivery, randomisation at the PHC level ensures that all participants at a given centre receive the same intervention conditions. This methodology strengthens the internal validity of the study while allowing for a pragmatic evaluation of how the intervention is implemented within existing clinical contexts ( Baumel *et al*., 2020).

Finally, another strength is the inclusion of partners as study participants. Fathers and non-birthing partners experience mental health difficulties at significant rates ( Leach *et al*., 2016; Rao *et al*., 2020) however remain largely overlooked in perinatal mental health interventions. By incorporating partner-specific content, the e-Perinatal intervention promotes a systemic view of family mental health, recognising the role of shared caregiving and dyadic support in improving outcomes for both parents and infants.

Despite these strengths, certain limitations must be acknowledged. First, the absence of recent prevalence studies on perinatal depression and anxiety in Spain poses challenges for accurately estimating the sample size. The most recent available studies were conducted during the COVID-19 pandemic ( Motrico *et al*., 2022), which may have inflated prevalence estimates due to the unique stressors of that period. Although the selected sample size aligns with established recommendations for pilot trials ( Eldridge *et al*., 2016a; Lancaster *et al*., 2004), the lack of updated national data may limit the precision of sample estimation.

Second, there is a scarcity of validated instruments specifically adapted for the Spanish perinatal population. Although this study uses internationally validated measures, some instruments have not been psychometrically validated in Spain for this population. Future research should prioritise the adaptation and psychometric validation of these instruments to ensure their reliability and validity in this context.

In conclusion, this pilot study represents a significant step toward the development and evaluation of an evidence-based, personalised digital intervention for the universal prevention of perinatal depression and anxiety. If the e-Perinatal intervention is shown to be feasible and acceptable, it could enhance perinatal mental health care by offering an accessible, cost-effective, and scalable complement to existing services. The findings from this study will contribute to the refinement of digital prevention strategies and inform future research and clinical practice aimed at improving maternal and paternal mental well-being during the perinatal period.

## Ethics and consent

All participants will provide written electronic informed consent prior to participation in the study. The research is conducted in accordance with the ethical principles of the Declaration of Helsinki.

Ethical approval was obtained from the Ethics Committee for Biomedical Research of the Hospitales Universitarios Virgen Macarena-Virgen del Rocío, affiliated with the Andalusian Health Service, Spain. The approval was granted under reference number SICEIA-2024-001659 on July 17, 2024.

## AI use disclosure

The authors acknowledge the use of generative AI tools to support the translation of this manuscript from Spanish to English. Specifically, OpenAI’s ChatGPT (version GPT-4o) was used to assist in refining the language and improving the clarity of the translated text. The authors assume full responsibility for the final content and have carefully verified its accuracy and appropriateness. In addition, a native English-speaking proofreader reviewed the final manuscript to ensure linguistic quality and consistency.

## Data Availability

This article is a study protocol. Therefore, no data are associated with it. This study follows the Standard Protocol Items: Recommendations for Interventional Trials (SPIRIT) guidelines ( Butcher *et al*., 2022; Chan *et al*., 2013). The completed SPIRIT checklist has been deposited in Zenodo ( Nieto-Casado *et al*., 2025). https://doi.org/10.5281/zenodo.15470066. Zenodo: SPIRIT checklist for “A Personalized mHealth Intervention for the Universal Prevention of Perinatal Mental Disorders in Routine Maternal Care (e-Perinatal): A Protocol Study for a Hybrid Feasibility Pilot Trial”. This project contains the following data: ePerinatal Protocol Pilot_SPIRIT Checklist.pdf Data are available under the terms of the Creative Commons Attribution 4.0 International license (CC-BY 4.0).
