# Supplementary material for: A personalized mHealth intervention for the universal prevention of perinatal mental disorders in routine maternal care (e-Perinatal): A protocol study for a hybrid feasibility pilot trial
Source: Open Res Eur. 2025 Jun 27;5:170. [Version 1] doi: 10.12688/openreseurope.20386.1 (PMC12402766; doi:10.12688/openreseurope.20386.1)
Supplement: Peer review history

# Reviewer report: Reviewer response for version 1

**DOI:** 10.21956/openreseurope.22063.r56893

**Published:** 2025-09-01

**Authors:** Vivek V. Nemane

**Affiliations:** 1 Symbiosis Law School, Symbiosis International (Deemed University), Pune, India

The reviewer acknowledges that the peer review process has undoubtedly provided an impetus to contribute towards the academic discourse which the paper seeks to advance.

 This is a well-structured hybrid type-1, two-arm cluster- Randomised Control Trial (RCT) pilot study testing “e-Perinatal app” within routine Primary Healthcare Centres (PHCs) in Andalusia, Spain, bearing registration (NCT06640907) and SPIRIT guidelines alignment.

 The protocol study discusses each step of the proposed pilot trial with notable precision. It presents a thorough scholarly literature and detailed description of research components. Despite the complexity of research technicalities, the information is presented in a clear and organized manner. The paper clearly depicts a step-by-step progression to adequately cover the ambit of the proposed pilot trial.

 The study proposes a very pragmatic target sample (n=96) and outcomes based on mixed-methods with variables including a) feasibility b) acceptability c) preliminary effectiveness (EPDS, GAD-7, MINI; NoMAD, ORIC; uMARS, Qualitative interviews). The intervention includes a) a micro intervention mHealth app that uses a rule-based filter and provides a forum for a psychologist-staffed chat with an organised training for Healthcare Professionals. The participants also receive structured educational emails as a resource. Data flow is premised on the REDCap and in-app captures. The analysis is based on a) feasibility assessment b) thematically coded interviews c) logistic models for incidence d) linear mixed effects for symptoms fulfilling the accountability towards Interclass Correlation Coefficients (ICCs).

 The protocol study is a well-crafted hybrid trial. Directed by the Normalization Process Theory, and embedded within routine PHC, it’ an inquiry into the implementation issues. It specifies primary outcomes with clarity and carefully articulates risk management. By resorting to the mHealth intervention, it opens the door for further studies in this area of contemporary importance. Furthermore, by highlighting the lack of empirical (field) research in this area, it encourages the integration of mHealth/digital approaches for the universal prevention of perinatal mental disorders in routine maternal care. The English language (as stated in the AI use disclosure, page 15) depicts precision, linguistic quality and consistency.

 Overall, the paper is well-written and doesn’t require substantial editing.

 While acknowledging the above-stated strengths, the reviewer sincerely hopes that following points will be helpful in enhancing the clarity and quality of the protocol study:

**Page 1 Title** is appropriate.

**Page 2 Abstract** can be expanded by adding one or two sentences – on methods used for data analysis.

**Page 2 Keywords** – The authors may want to add ‘protocol study’.

**Introduction (Page 4)** –  For “high dropout rates and limited integration into routine clinical practice”, the manuscript details - a few procedural measures as part of the study protocol. The authors should describe specific **‘reasons’** for these issues. This will help in preventing similar issues during the proposed pilot trial.

**Introduction (Page 4 Para 7)** – This para discusses advantages of mHealth interventions. Authors should highlight studies that identify problems/barriers relating to the mHealth interventions to present **holistic view** to the readers.

**Study Setting (Page 5)** – Specify sample size targets and details on variations in sampling (regarding urban vs. rural PHCs, mHealth app use intensity).

**PHC and participants (Page 5)** - For mHealth interventions, at the participant level, eligibility for women include: d) access to the mobile phone and to internet and f) have a personal email account. Here, the authors should

 a) highlight any studies that confirm these points as a criterion to validate the efficient mobile literacy OR

 b) clearly articulate the presumption of mobile literacy amongst the participants OR

 c) include a separate criterion point to check the mobile literacy of the participants.

**PHC and participants (Page 5)** – One of the key distinguishing aspect of the study protocol is the inclusion of “partners”. The paper defines “partners” as the individual identified by the participant woman as her main support during the perinatal period. The paper also provides adequate description on “partners” but this requires further clarification since – the meaning of partners - ‘individuals as women’s main support’ is temporal dimension which may change over the time period. Within the context of law, there may be an overlap regarding the meaning of ‘partners’ as per the applicable laws/regulations in the jurisdiction.

 Also, since partners are considered as ‘optional’ in the study, authors should specify whether data relating to partners will be analysed purely at an individual level or will it be a part of interactions and influences between two individuals? Here, authors should specify their co-relation with the outcome measures.

**Intervention arm (Page 6)** – The paper states that the base content of the e-Perinatal app includes “psychoeducational materials, psychological strategies, mindfulness practices, and physical activity resources, all tailored to the needs of pregnant and postpartum women and their partners”. The authors should justify the relevance of this content by providing adequate references of previous studies or publications in this area.

**Intervention arm (Page 6)** – With reference to Digital micro-interventions (DM), are there any previous studies that espouse the ‘effectiveness’ of chosen DMs?

**Intervention arm (Page 6)** – Provide a clear justification for the clinical rule-based filtering algorithm with further clarification on algorithmic accountability.

**Eligibility of Healthcare professionals and the eligibility of the dedicated recruitment coordinator (Page 6) **– The authors should consider adding pre/post training knowledge and confidence scales for healthcare professionals. This can be linked to the fidelity and adoption related outcomes.

**Control arm (Page 6)** – As per the manuscript - participants in the control arm are supposed to receive monthly emails containing educational resources on perinatal mental health, pregnancy, and infant development. The manuscript further adds that “These materials, developed and publicly distributed by the Andalusian Health Service, provide general information without personalised content”.

- For transparency purposes, these emails can be archived as non-personalized material.

**Economic data (Page 10)** – As per the manuscript, “economic data will be obtained through a telephone interview at t1”.

- The authors should specify a) a recall window b) pilot costing instruments

**Table 2 (Page 12)** –

 a) Specify precise duration/window of duration in terms of days for t1/t2                                     

 b) Specify precise classification of windows (as app-based or REDCap)

**Strategies for Trail Retention and Engagement (Page 13)** – To maximise participant retention and engagement, current plan is to provide gifts and reminders. Regarding engagement with the physical activity components of the e-Perinatal app, the authors may consider adding - engagement triggers and engagement metrics.

**Data collection and management (Page 13)** – The reviewer is concerned regarding the data collection and management plan. Please see the comment about this below:

 The authors should clearly specify the data protection regulations. The paper mentions regarding the use of secure study database and REDCap which is a secure electronic data capture platform. The authors have articulated and explicitly talked about privacy concerns as impediments to mHealth implementation. It’s important to address this concern thoroughly considering the sensitivity of data protection issues. The authors should consider adding a data-flow diagram that clearly depicts the flow of data from device to server (including the details regarding):

 1. transport and at-rest encryption

 2. Access control

 3. data retention and data deletion timelines

 4. policy/plan to moderate a discussion forum or a chat function

 5. policy/plan to address the incursion of adverse or unnecessary content

 6. location of app server hosts

 7. applicable law/regulations governing the data processing.

 The authors should consider adding a separate data management plan which clearly articulates the above-stated details.

**Potential risks for participants (Page 13)** – The manuscripts states that - the app poses minimal risk, even for users who interact with the content with limited interest. The authors should discuss the ‘minimal risks (For e.g. any adverse event related to mental health).

**Dissemination (Page 14)** – The paper states that “All datasets and documentation resulting from this study will be made openly available via the Zenodo repository”.

- The authors should clearly specify the data that will be shared including de-identification procedures and restricted access exceptions.

**Specific comments pursuant to methodology:**

- The authors should clearly define the fidelity metrics and a strategy to address any deviations, in case needed.
- The authors should consider providing clear and explicit quantitative thresholds for feasibility and acceptability benchmarks. The benchmarks will help in deciding future trial parameters (for e.g. cluster size).
- The authors should specify cluster size and anticipated range of Interclass correlation coefficients (ICCs). This will help in justifying the precision of feasibility metrics. The current sample target (n=96) is pragmatic but sample for intervention per arm and intervention per cluster lacks precision.
- In this study, the clusters will be represented by the primary healthcare centres (PHC). The randomization will be implemented using sealed envelopes and by blinding the MINI interviewer. Here, the details can be added on a) sequence generation (for e.g. based on random block sizes? Or based on rural/urban stratification?) Also, there may be instances requiring a separate monitoring strategy. This includes for e.g. staff movement within PHCs, participant forum access being restricted to intervention PHCs.
- The authors have proposed MINI International Neuropsychiatric Interview to measure the cumulative incidence of perinatal depression and anxiety among pregnant and postpartum women. The authors should provide a justification to support the proposed method.
- The authors have listed primary, secondary and exploratory outcome measures. These include for e.g. infant, sleep, fitness, mood, experiences of satisfaction and frustration of the basic psychological needs, breastfeeding). Here, the secondary outcomes (on page 7) and other outcomes (page 9) are overlapping to the extent. Authors may want to create ‘a core secondary set’ and move rest of the outcome measures to the ‘exploratory’ outcome measures. The authors can also depict the mapping of each outcome to a decision criterion in a separate table.

The reviewer trusts that careful consideration of the above-stated observations will offer insights in further refining the paper that deals with a public health issue of significant importance.

Is the study design appropriate for the research question?

Yes

Is the rationale for, and objectives of, the study clearly described?

Yes

Are sufficient details of the methods provided to allow replication by others?

Partly

Are the datasets clearly presented in a useable and accessible format?

Yes

Reviewer Expertise:

-

I confirm that I have read this submission and believe that I have an appropriate level of expertise to confirm that it is of an acceptable scientific standard, however I have significant reservations, as outlined above.

# Reviewer report: Reviewer response for version 1

**DOI:** 10.21956/openreseurope.22063.r56894

**Published:** 2025-08-23

**Authors:** Ritika Behl

**Affiliations:** 1 School of Legal Studies and Governance, Vidyashilp University, Bengaluru, India

**Comments & Feedback for the Authors**

  

1. Abstract: -  

Introduction: -

 During the perinatal period, new-onset of depression as well as relapse is witnessed, and is well-reported by the existing literature. My request would be to emphasize both aspects.

1. It is not merely structural barriers that result in high treatment gaps and low service demand for perinatal mental disorders. These barriers also include individual-level, healthcare system-level, societal-level, and organizational-level barriers. I suggest not limiting the barriers to just one category.

The above-stated suggestions also apply to the Introduction section of the manuscript.

  

1. Introduction: -

1. It is essential to highlight why universal targeted interventions must be introduced at the Primary Care level itself. Please incorporate a brief text in this regard to make this work even more relevant and necessary.

 

1. Protocol: -

1. Please provide details about the ethical approval and funding after the trial registration details.
2. I would like you to clarify why postpartum women only up to 5 months have been included in the eligibility criteria. In general, the postpartum period is accepted to last from childbirth to 12 months postpartum. Why have you deviated from the general understanding when the app can be accessible to women beyond 5 months as well? Is it related to the existing care pathways? Please clarify this aspect.
3. Who will check on eligibility at the PHC, participant, and partner levels, which author, or someone not part of the author team? Please include these details as well. How will informed consent be taken and recorded?
4.  Also, please provide further details about the randomization process. Again, who will do it, and how will they do it? Will the participants be blind to the process? Explain it, even though briefly.

 

1. Intervention: -

1. Provide details about the author/s who will be undertaking the specialized training for professionals in the intervention arm.

 

 Outcomes: -

1. Kindly clarify why two different timepoints, including at the time of enrollment, and one day after enrollment, which you call as baseline, have been selected? Shouldn’t there be only one time point as baseline, whether you select it as ‘at the time of enrollment’ or ‘one day later’?

 

1. Please move the following sections, including ‘Participant timeline’, ‘Sample Size’, ‘Recruitment’, and ‘Allocation’, before the ‘Outcomes’ section.
2. Please move the ‘Potential risk for participants’ section after the ‘Intervention’ section.
3. Please provide details about the informed consent taken from the participants in the ‘participants’ section only.
4. I also suggest providing details about the ‘Reporting guidelines’ in the ‘Protocol’ section.

Is the study design appropriate for the research question?

Yes

Is the rationale for, and objectives of, the study clearly described?

Yes

Are sufficient details of the methods provided to allow replication by others?

Partly

Are the datasets clearly presented in a useable and accessible format?

Yes

Reviewer Expertise:

Perinatal Mental Health, Women's Mental Health, Health Laws and Policies, Human Rights

I confirm that I have read this submission and believe that I have an appropriate level of expertise to confirm that it is of an acceptable scientific standard, however I have significant reservations, as outlined above.

# Reviewer report: Reviewer response for version 1

**DOI:** 10.21956/openreseurope.22063.r56245

**Published:** 2025-08-23

**Authors:** Isabel F. Almeida

**Affiliations:** 1 University of California, Irvine, USA

The introduction outlines the significant impact of perinatal mental disorders (PMD) on parents, families, and children, highlighting their prevalence and consequences. It discusses the potential benefits of universal preventive interventions to address PMD, emphasizing digital solutions (mHealth apps) to overcome existing barriers in healthcare systems. The introduction identifies current gaps, including limited partner involvement and insufficient empirical support for many existing interventions, and suggests using structured frameworks for effective implementation. The study aims to test the feasibility, acceptability, and preliminary effectiveness of the e-Perinatal app in routine care settings.

 The introduction clearly establishes the importance of perinatal mental health, effectively outlining prevalence rates and consequences for families and society.

 Researchers presented existing gaps in perinatal mental health care and prevention, providing strong justification for the study.

 Integration of theoretical frameworks grounds study within established approaches.

 The introduction could be more concise by combining some references that support similar points.

 Researchers may clarify how intervention specifically addresses key limitations of existing mHealth solutions (e.g., partner involvement, professional guidance, privacy concerns).

 Authors may consider briefly mentioning why Andalusia, Spain, is a particularly relevant setting, making the context-specific need clearer.

 What is meant by "universal preventive interventions" in simple terms?

 The study design and methods is clear and transparent.

 There is a clear justification for sample size  appropriate for a pilot trial.

 The rationale on why choosing specifically the eight primary healthcare centers could be briefly emphasized (e.g., diversity in socioeconomic status, rural/urban mix).

 Given that partner participation is voluntary, how might differences in partner involvement influence the effectiveness of the intervention, and how might researchers measure or interpret these variations? This can be added to the discussion. 

 Overall, a well-written report on a subject of public health importance.

Is the study design appropriate for the research question?

Yes

Is the rationale for, and objectives of, the study clearly described?

Yes

Are sufficient details of the methods provided to allow replication by others?

Yes

Are the datasets clearly presented in a useable and accessible format?

Yes

Reviewer Expertise:

Maternal/Child Health

I confirm that I have read this submission and believe that I have an appropriate level of expertise to confirm that it is of an acceptable scientific standard.
